# The global regulator SpoVG modulates *Staphylococcus aureus* virulence through Agr-dependent pathways

**DOI:** 10.1080/21505594.2025.2561827

**Published:** 2025-09-15

**Authors:** Kai Ma, Li Xu, Sijing Zhang, Ziheng Wu, Hui Wang, Ting Xue

**Affiliations:** a School of Life Sciences, Anhui Agricultural University, Hefei, Anhui, China; b Division of Life Sciences and Medicine, University of Science and Technology of China, Hefei, Anhui, China; c Joint Research Center for Food Nutrition and Health of IHM, Anhui Agricultural University, Hefei, China

**Keywords:** *Staphylococcus aureus*, SpoVG, Agr system, virulence, hemolysis

## Abstract

*Staphylococcus aureus* (*S. aureus*) is a major pathogen responsible for a wide range of infections in both community and healthcare settings. Its virulence is orchestrated by a complex regulatory network. SpoVG, a global regulator in *S. aureus*, has been shown to play crucial roles in antibiotic tolerance, stress adaptation, and biofilm formation. However, its specific role in virulence regulation has remained poorly understood. Here, we investigated the role of SpoVG in the modulation of *S. aureus* virulence. The results demonstrate that deletion of *spoVG* significantly increased the hemolytic activity and virulence of the SA3 strain, as evidenced by both in vitro assays and murine infection models. Transcriptomic profiling and RT – qPCR analyses revealed that the inactivation of *spoVG* activated the Agr quorum-sensing system, resulting in upregulating key virulence genes, including *psmα*, *psmβ*, *hla*, and *hlg*. Further, β-galactosidase reporter assays and electrophoretic mobility shift assays (EMSA) confirmed that SpoVG directly binds to the promoter regions of *agrACDB*, *psmα*, and *psmβ*, thereby repressing their transcription. Collectively, these findings identify SpoVG as a critical negative regulator of virulence in *S. aureus*, acting through direct repression of the Agr system and its downstream effectors. This study provides new mechanistic insights into SpoVG-mediated virulence regulation and highlights SpoVG as a potential target for anti-virulence strategies against *S. aureus* infections.

## Introduction

*Staphylococcus aureus* is a major zoonotic pathogen capable of colonizing diverse host niches and causing a wide spectrum of infections, ranging from mild skin lesions to life-threatening diseases [1,2]. Its pathogenicity largely relies on a wide array of virulence factors that promote tissue adhesion and inflict damage through multiple mechanisms [3,4]. *S. aureus* infection is orchestrated by the coordinated expression of these virulence determinants. *S. aureus* employs a wide array of virulence factors whose coordinated expression facilitates successful colonization, immune evasion, and tissue invasion. Among these, α-toxin (Hla) is recognized as a key cytotoxin that disrupts epithelial barriers and promotes systemic dissemination through metalloproteinase-dependent cleavage of E-cadherin [5,6]. Beyond invasive infections, *S. aureus* also contributes to inflammatory skin diseases such as atopic dermatitis, in part by activating mast cells via secreted toxins including δ-toxin and phenol-soluble modulins (PSMs). Notably, PSMα peptides exhibit strong cytolytic activity against a variety of host cell types, thereby playing a critical role in both skin inflammation and immune modulation [7–9]. The complex regulatory network strictly regulates the expression of these virulence factors, at the core of which lies the accessory gene regulator (*agr*) quorum-sensing system [10,11].

Agr system is a crucial quorum-sensing system, regulating both physiological functions and pathogenicity in a cell-density-dependent manner, in *S. aureus* [12]. The Agr system is a global regulator composed of two opposing transcripts, RNAII and RNAIII, which are transcribed under the control of the P2 and P3 promoters, respectively [13]. The RNAII transcript encodes the two-component systems (TCS, AgrA, and AgrC) and the quorum-sensing elements (AgrD and AgrB). The precursor of the autoinducing peptide (AIP) is produced by AgrD, which is processed and transported to the extracellular space by the signal peptidase SpsB and the chaperone protein AgrB, forming the mature AIP. Once the extracellular AIP reaches a threshold concentration, it binds to and induces the histidine kinase AgrC on the cell membrane to autophosphorylate. This event triggers a cascade of intracellular signaling, transferring the phosphate group to the effector AgrA and then promoting the transcription of RNAII and RNAIII by binding to the p2 and p3 promoters [13,14]. RNAIII, a regulatory small RNA with a complex secondary structure, also encodes the δ-hemolysin. RNAIII is the primary effector molecule of the Agr system. It regulates the translation efficiency of target genes by binding to their mRNA sequences, thereby controlling the expression of virulence factors such as α-hemolysin (encoded by *hla*), and γ-hemolysin (encoded by *hlgABC*) [15]. In addition, AgrA directly binds to the promoter of phenol-soluble modulins (PSMs) to activate the PSMs, independently of RNAIII [16].

Stage V sporulation protein G (SpoVG) is a multifunctional regulator in Gram-positive bacteria, contributing to processes such as sporulation, antibiotic resistance, and virulence regulation [17–19]. In *S. aureus*, SpoVG has been reported to control the transcription of virulence factors including *spa* and *clfB*, modulating the pathogenicity of the model strain N315 [20]. However, the N315 strain naturally lacks a functional *agr* system, suggesting that its virulence regulation may not reflect that of most *S. aureus* strains.

Here, we demonstrate that deletion of *spoVG* significantly enhances hemolytic activity, while its overexpression markedly suppresses this phenotype. Transcriptomic profiling and RT-qPCR analyses revealed that *spoVG* deletion leads to upregulation of key virulence genes (*hla*, *psm*, and *hld*) along with the Agr quorum-sensing system. EMSA confirmed that SpoVG directly binds to the P2 promoter of the agr operon. Strikingly, hemolytic activity was substantially attenuated in the *spoVG agr* double mutant relative to the SA3 strain. Consistently, in a murine skin infection model, the *spoVG* mutant induced significantly larger abscesses, whereas virulence was markedly reduced in the double mutant. Together, our findings establish SpoVG as a negative regulator of Agr-dependent virulence, constraining pathogenicity in *S. aureus*.

## Materials and methods

### Bacterial strains, plasmids, and growth conditions

All bacterial strains and plasmids used are listed in Table 1. *Escherichia coli* (*E. coli*) were cultured in Luria–Bertani (LB) medium (Oxoid), and *S. aureus* in tryptic soy broth (TSB) (Difco). When appropriate, add antibiotics to select and maintain the plasmid, with the final concentrations: chloramphenicol (Cm); 15 μg/mL and ampicillin (Amp), 100 μg/mL.

**Table 1.** Strains and plasmids were used in this study.

Strains or plasmids	Description	Reference or source
Strains		
*E. coli*		
DH5α	Clone host strain	TransGen
BL21(DE3)	Expression strain	TransGen
*S. aureus*		
SA3(WT)	isolated from dairy cows with mastitis	[21]
RN4220	8325–4, restriction-negative strain	NARSA
WTΔ*spoVG*	SA3 *spoVG*-deletion mutant	This study
WTΔ*agr*	SA3 *agr*-deletion mutant	This study
Δ*spoVG*Δ*agr*	SA3 *spoVG*-deletion and *agr*-deletion double mutant	This study
WT/pRMC2	WT with the empty vector pRMC2 Amp^r^, Cm^r^	This study
Δ*agr*Δ*spoVG*/pRMC2	Δ*spoVG*Δ*agr* with the empty vector pRMC2 Amp^r^, Cm^r^	This study
Δ*agr*Δ*spoVG*/pRMC*spoVG*	Δ*spoVG*Δ*agr* with the complement plasmid pRMC-spoVG Amp^r^, Cm^r^	This study
Δ*agr*Δ*spoVG*/pRMC*agr*	Δ*spoVG*Δ*agr* with the complement plasmid pRMC-agr Amp^r^, Cm^r^	This study
WT/pLI50	WT with the empty vector pLI50 Amp^r^, Cm^r^	This study
Δ*spoVG*/pLI50	WTΔ*spoVG* with the empty vector pLI50 Amp^r^, Cm^r^	This study
C*spoVG*	WTΔ*spoVG* with the complement plasmid pCspoVG Amp^r^, Cm^r^	This study
SH1000	8325–4, rsbU repaired	NARSA
SH1000/pLI50	SH1000 with the empty vector pLI50 Amp^r^, Cm^r^	This study
SH/Δ*spoVG*	SH1000 *spoVG*-deletion mutant	This study
SH/Δ*spoVG*/pLI50	SH/Δ*spoVG* with the empty vector pLI50 Amp^r^, Cm^r^	This study
SHC*spoVG*	SH/Δ*spoVG* with the complement plasmid pCspoVG Amp^r^, Cm^r^	This study
Plasmids		
pBTs	Shuttle vector, temp sensitive, Amp^r^ Cm^r^	[22]
pBT-*spoVG*	pBTs derivative, for *spoVG* mutagenesis; Amp^r^, Cm^r^	This study
pBT-*agr*	pBTs derivative, for *agr* mutagenesis; Amp^r^, Cm^r^	This study
pLI50	Shuttle vector, Amp^r^, Cm^r^	Novagen
pC*spoVG*	pLI50 with *spoVG*	This study
pOS1	shuttle vector, Amp^r^, Cm^r^	[23]
pOSpsmα	pOS1 derivative, harboring ORF of *psmα* and its promoter, Amp^r^, Cm^r^	This study
pOSpsmβ	pOS1 derivative, harboring ORF of *psmβ* and its promoter, Amp^r^, Cm^r^	This study
pOShla	pOS1 derivative, harboring ORF of *hla* and its promoter, Amp^r^, Cm^r^	This study
pOShlg	pOS1 derivative, harboring ORF of *hlg* and its promoter, Amp^r^, Cm^r^	This study
pOSagr	pOS1 derivative, harboring ORF of *agr* and its promoter, Ampr, Cmr	This study
pOSRNAIII	pOS1 derivative, harboring ORF of *hld* and its promoter, Amp^r^, Cm^r^	This study
pRMC2	shuttle vector, ATC inducible promoter, Amp^r^, Cm^r^	[24]
pRMC*spoVG*	pRMC2 derivative, harboring ORF of *spoVG*, Amp^r^, Cm^r^	This study
pRMC*agr*	pRMC2 derivative, harboring ORF of *agrBDAC*, Amp^r^, Cm^r^	This study

Amp^r^, ampicillin-resistant; Cm^r^, chloramphenicol-resistant.

### Construction of the spoVG single mutation and spoVG, agr double mutation

*E. coli* DH5α was employed as a cloning host for plasmid construction. Gene mutations were based on the principle of homologous recombination, generated using the plasmid pBTs, as previously described [22]. The upstream and downstream homologous regions of the target genes were amplified by PCR and subsequently cloned into the pBT plasmid. The recombinant plasmid was modified with *S. aureus* RN4220, followed by transformation into the target strains. Allelic replacement mutants were selected and confirmed by PCR and sequencing. Primer sequences used in this study are listed in Table 2.

**Table 2.** Primers for this study.

Primer name	Oligonucleotide (5′-3′)	Reference or source
agr-up-kpnI-F	CGGAATTCGAGCTCGGTACCCATTATGGGATAACGCTGAA	This study
agr-up-R	AGGTCTTAGCTCGAAAGAGCGAATTGTCTA	
agr-down-F	GCTCTTTCGAGCTAAGACCTGCATCCCTAA	This study
agr-down-SalI-R	GCATGCCTGCAGGTCGACAACTCAGTAAGAACCCATTT	
spoVG-up-kpnI-F	GCGGGTACCAATTGCCAGTATTTACATGG	This study
spoVG-up-R	TACATCGCTACCCCCCTATAGTATATATCTC	This study
spoVG-down-F	TATAGGGGGGTAGCGATGTAATACATTTGC	This study
spoVG-down-SalI-R	CGCGTCGACTATTCACCTTGCGCATTATC	This study
Check-pBT-F	TCACCGACAAACAACAGA	[22]
Check-pBT-R	CCAAGCCTATGCCTACA	
Check-agr-F	TTTTATCGTAAGCCCTCTGC	This study
Check-agr-R	GTGCCATTGAAATCACTCCT	
Check-spoVG-F	CGCTGATGTTCAAGCACAGA	This study
Check-spoVG-R	AAAGAACGCTCGCCTAAATG	This study
pLI-*spoVG*-kpnI-F	GCGGGTACCAATTGCCAGTATTTACATGG	This study
pLI-*spoVG*-SalI-R	CGCGTCGACTATTCACCTTGCGCATTATC	
pLI50-F	CCTGACGTCTAAGAAACCAT	This study
pLI50-R	CGATAACCACATAACAGTCA	
pET-spoVG-F	CATGCCATGGGAATGAAAGTGACAGATGTAAGAC	This study
pET-spoVG-R	CCGCTCGAGAGCTTCTTCTGAATCTTCTGATGTAGC	This study
pRMC-spoVG-F	AGATCTGGGAGGCCGTTTC CAGAGAGATATATACTATAGG	This study
pRMC-spoVG-R	CGGCCAGTGAATTCGAGCTC TGTATTACATCGCTAAATAT	This study
pRMC-agr-F	AGATCTGGGAGGCCGTTTCTTGAATTATTTTGATAATAAAATTG	This study
pRMC-agr-R	CGGCCAGTGAATTCGAGCTCTTATTATATTTTTTTAACGTTTCT	This study
rt-*hu*-F	AAAGAAGCTGGTTCAGCAGTAG	[25]
rt-*hu*-R	TTTACGTGCAGCACGTTCAC	
rt-psmα3-F	TTCGTAGCAAAATTATTC	This study
rt-psmα3-R	ATGAGTTGTTGATCGTTA	This study
rt-psmβ1-F	CTAGTAAACCCACACCGTTA	This study
rt-psmβ1-R	TATTTCAAAGGTGAGGGA	This study
rt-psmβ2-F	AGAATCCGAATAATTTACC	This study
rt-psmβ2-R	TAAAGATACCGTAACTGC	This study
rt-hla-F	AAAGTAGGCTGGAAAGTGAT	This study
rt-hla-R	GTAGCGAAGTCTGGTGAAAA	This study
rt-hlgB-F	TTTGTCTGCCAGCTAAGAAG	This study
rt-hlgB-R	AATGGTTTATCTGGTGGACT	This study
rt-hlgA-F	CCATGTTTCTGCCGTAAGTG	This study
rt-hlgA-R	AACTGGTCCAGCAGCAAGAG	This study
rt-hld-F	GGCACAAGATATCATTTC	This study
rt-hld-R	ACTATACGAAGATAACAA	This study
rt-agrA-F	ACGAATTTCACTGCCTAA	This study
rt-agrA-R	TTCATTTGCGAAGACGAT	This study
rt-agrC-F	TGATAGACCTAAACCACGAC	This study
rt-agrC-R	ACCCGATGAAGTAAGTAGC	This study
rt-agrD-F	ACTTCAACTTCATCCATT	This study
rt-agrD-R	TTGATTTTATTACTGGGA	This study
rt-agrB-F	CAAATGGCTCTTTGATGA	This study
rt-agrB-R	AAAGAAGCCCATTCCTGT	This study
pOS-psmα-F	TAAAGACGATCCGGGGAATTCTGCATAACCTCCTTATTTC	This study
pOS-psmα-R	GTTGTAAAACGACGGGATCCGGGCCAGCGATGATACCCAT	This study
pOS-psmβ-F	TAAAGACGATCCGGGGAATTCCACGTTTAACAACACAAGA	This study
pOS-psmβ-R	GTTGTAAAACGACGGGATCCGGGTTAAATAAACCTTCCATTG	This study
pOS-hla-F	TAAAGACGATCCGGGGAATTCTTTAATCCCCTATCATATTT	This study
pOS-hla-R	GTTGTAAAACGACGGGATCCGGGACTATACGTGTTTTCAT	This study
pOS-hlg-F	TAAAGACGATCCGGGGAATTCATAATCAATCAAAAGTATC	This study
pOS-hlg-R	GTTGTAAAACGACGGGATCCGGTATTTTATTTTTAATCAT	This study
pOS-agr-F	TAAAGACGATCCGGGGAATTCATGTTAAAATATTAAATACAAATTAC	This study
pOS-agr-R	GTTGTAAAACGACGGGATCCGGATTATCAAAATAATTCAA	This study
pOS-RNAIII-F	TAAAGACGATCCGGGGAATTCGTAATTTGTATTTAATATTTTAA	This study
pOS-RNAIII-R	GTTGTAAAACGACGGGATCCGGAATGATATCTTGTGCCAT	This study
P-psmα-F	ACCTCCTTATTTCTAATCTCT	This study
P-psmα-R	AGATTACCTCCTTTGCTT	This study
P-psmβ-F	TGAAAACACTCCTTAAAATT	This study
P-psmβ-R	CACGTTTAACAACACAAGAA	This study
P-agr-F	TTTTACACCACTCTCCTCAC	This study
P-agr-R	TGCCATTGAAATCACTCCTT	This study
P-hla-F	TTTAATCCCCTATCATA	This study
P-hla-R	TTTCATCATCCTTCTAT	This study
P-hlg-F	AGAAATCACTTTCTTTCTATT	This study
P-hlg-R	TCATTCTGGAAATAATCAATC	This study
*hu*-F	AAAGAAGCTGGTTCAGCAGTAG	This study
*hu*-R	TTTACGTGCAGCACGTTCAC	This study

The sequences underlined refer to the restriction endonuclease recognition sites.

### Construction of complementation and overexpression strains

Plasmids pLI50 and pRMC2 were employed for gene complementation and overexpression. For functional complementation of the *spoVG* mutant, the *spoVG* gene along with its native promoter was amplified from the SA3 strain using primers pLI-*spoVG-Kpn* I-F and pLI-*spoVG-Sal* I-R, and cloned into the shuttle vector pLI50 to generate pC*spoVG*. Similarly, for functional complementation of the *agrBDAC* mutant, we used the ATC-inducible plasmid pRMC2. The *agrBDAC* gene was amplified from the SA3 strain using primers pRMC-*agr*-F and pRMC-*agr*-R and cloned into the shuttle vector pRMC2 to generate pRMC-*agr*. To construct the overexpression plasmid pRMC*spoVG*, the *spoVG* ORF was amplified from SA3 genomic DNA using primers pRMC-*spoVG*-F and pRMC-*spoVG*-R, and inserted into pRMC2. All recombinant plasmids were first introduced into *S. aureus* RN4220 for restriction modification and then transformed into both wild-type (WT), *spoVG* mutant strains, and Δ*spoVG*Δ*agr* double mutant strains to generate the complementation and overexpression strains.

### Determination of haemolytic activity

The bacteria cultured overnight was adjusted to the OD_600_ = 1.0 and take 2 μL of bacterial solution and dropped onto the sheep blood agar plate, incubated at 37°C for 24 h, and the size of the hemolytic ring was measured. The quantitative detection of hemolytic activity is by the method of co-culturing the sample with off-fiber sheep blood. Briefly, centrifuging the overnight culture, taking 100 μL supernatants mixed to 900 μL phosphate-buffered saline (PBS) containing 5% off fiber sheep blood, incubating in 37°C for 30 min, and measuring the absorbance value of the supernatant at 543 nm after centrifugation at 10,000 rpm/min for 2 min [23]. The ddH_2_O treatment group was used as the positive control, and the PBS treatment group was used as the negative control. The positive control was regarded as 100% hemolysis rate, and we calculated the percentage hemolysis for each sample.

### RNA extraction and sequencing

The *S. aureus* SA3 and Δ*spoVG* cultures were collected for RNA extraction, respectively. Biozeron Biotechnology Co., Ltd. helps us with RNA sequencing and library construction, and provides high-quality reads for sequence analysis and bioinformatics data analysis. Refer to Wang et al. for a description of the method [26]. Briefly, the cultures of each strain during the exponential growth phase (OD_600_ = 1.5) were collected, and the total RNA was extracted using the TRlzol method. Illumina TruSeq RNA Sample Preparation Kit (San Diego, CA) was used to prepare RNA-Seq strand-specific libraries. Then, after rRNA removal, cDNA synthesis, and end repair, a 200–300 bp cDNA library was selected and sequenced using Illumina NovaSeq 6000 (150 bp × 2, Shanghai BIOZERON Co., Ltd).

### Total RNA extraction and quantitative real-time PCR

Overnight *S. aureus* cultures were diluted to an OD_600_ = 0.03 in fresh TSB, and shaking culture was continued to collect cells in the different growth periods (OD_600_ were 0.5, 1.5, 3.0, 5.0) by centrifugation at 10,000 × g, and then cells were resuspended with RNase-free water containing 10 mg/mL lysozyme and 40 µg/mL lysostaphin (both from Sangon) and incubated at 37°C for 1 h to lyse the cells. After incubation, total RNA was extracted from cells using the Trizol method. Removal of gDNA and synthesis of cDNA were performed using the EasyScript One-Step gDNA Removal and cDNA Synthesis SuperMix kit [TransGen Biotech (Beijing) Co. Ltd.]. *hu* served as an internal reference gene to normalize the abundance of the target genes [25].

### β-galactosidase activity assay

The promoters of *agr*, *hla*, *hlg*, *psmα*, and *psmβ* were ligated to the promoter-free lacZ plasmid pOS1 via homologous recombination, resulting in the construction of a transcriptional LacZ fusion reporter plasmid. Subsequently, the plasmids were transferred into WT and *spoVG* mutant strains by electroporation for β-galactosidase activity detection. The WT and *spoVG* mutant strains containing different reporter plasmids were incubated overnight and then transferred to fresh 50 mL TSB medium containing chloromycetin for 4 h of shaking culture, and then collected bacteria for enzyme activity assay. Resuspend the collected cells separately with ABT (0.1% (V/V) Triton X-100, 100 mM NaCl, 60 mM K_2_HPO_4_, 40 mM KH_2_PO_4_) and incubate at 37°C until thoroughly lysed. Then add 100 μL ABT and 4 mg/mL o-nitrophenyl-β-D galactopyranoside (ONPG) to start the reaction. Incubate at 37°C until the solution turns yellow and record the reaction time, then add 1 mL of Na_2_CO_3_ (1 M) to terminate the reaction. The absorbance of the sample at 420 nm was measured and calculated the enzyme activity using the following formula: units = (1000 × OD_420_)/(T × V × OD_600_).

### SpoVG protein expression and purification

The C-terminal His_6_-tag SpoVG protein was produced as previously reported [27]. The *spoVG* gene from SA3 was PCR-amplified using primers pET-*spoVG*-F and pET-*spoVG*-R, and cloned into the pET28a(+) vector. The resulting plasmid, pET-spoVG, was validated by PCR with T7 primers and confirmed by sequencing. It was introduced into *E. coli* BL21(DE3) via chemical transformation. Protein expression was induced with 0.5 mM IPTG at 16°C 150 rpm/min for 12 h, and the His₆-tagged SpoVG protein was isolated using a HisTrap affinity column. SDS-PAGE was used to verify purity, and protein concentration was measured using a BCA assay (Beyotime).

### Electrophoretic mobility shift assay (EMSA)

Promoter-containing DNA fragments of target genes were amplified from SA3 genomic DNA, separated by agarose gel electrophoresis, and subsequently purified. Varying amounts of protein were incubated with the DNA probe at room temperature for 30 min. The mixtures were then loaded onto a 6% native polyacrylamide gel and subjected to electrophoresis in 0.5 × Tris-borate-EDTA (TBE) buffer at 120 V for 80 min. The gel was immersed in a TBE buffer containing nucleic acid dye for 5 min, and then the band shifts were detected by UV mode of the gel imaging system [28]. Moreover, previous research has established that SpoVG positively regulates the synthesis of capsular polysaccharides in *S. aureus* [29], so the *cap* promoter was used as a positive control for EMSA experiments.

### Mouse skin infection model

Six-week-old outbred, immunocompetent female BALB/c mice were obtained from Henan SKBEX Biotechnology Co., Ltd. and maintained under specific pathogen-free (SPF) conditions in individual cages. Skin infection experiments were performed after 1 week of rearing, and before infection, the dorsal hair was shaved with a razor prior and the mice were labeled by ear clipping. After 4 h of *S. aureus* shaking culture, the viable count was determined by serial dilution plating to establish the infectious dose. Anesthetize mice by intraperitoneal injection of 1% pentobarbital sodium (50 mg/kg), and after anesthesia, 50 μL of PBS containing 2.5 × 10^8^ live *S. aureus* are injected subcutaneously. Each group contained eight randomized mice, with six groups in total (*n* = 48), and a PBS-injected group served as the control. The experiment lasted for 7 d, and the lesion area was calculated as maximal length × maximal width, following a previously reported method [23]. After the experiment, the mice were euthanized using cervical dislocation. The skin lesion site was all excised, and the tissue was homogenized in PBS, dilute and coat it on a plate to calculate the number of surviving *S. aureus* at the cyst site. Pathological analysis is required, the lesion site is excised and soaked in paraformaldehyde solution, paraffin-embedded sections are performed, followed by H&E (hematoxylin and eosin) staining and microscopic examination. The animal experiments were conducted between September and October 2024.

### Ethics statement

The animal use protocol listed has been reviewed and approved by the Institutional Animal Care and Use Committee of the Anhui Agriculture University (AHAUB2024010). We have adhered to ARRIVE guidelines and uploaded the checklist. Intraperitoneal injection of sodium pentobarbital (50 mg/kg) was used for anesthesia, and experiments were carried out after anesthesia. After the end of the experiment, the cervical dislocation method was used, and the cervical spine of the mouse was quickly dislocated with a special tool, so that the mouse immediately lost consciousness and stopped breathing. All procedures were carried out in compliance with the Regulations for the Administration of Affairs Concerning Experimental Animals as mandated by the State Council of the People’s Republic of China.

### Statistical analysis

All experiments were performed in biological triplicates. Graphing and analysis were performed using GraphPad Prism 8.0 (GraphPad Software Inc., GraphPad Prism 8.0.2, San Diego, CA, USA, 2018). Unpaired two-tailed *t* tests for equal or unequal variance were then performed to calculate the significant differences (*p* values), not significant (*p* > 0.05); *, *p* < 0.05; **, *p* < 0.01; ***, *p* < 0.001.

## Results

### SpoVG inhibited the haemolytic activity of *S. aureus*

**Figure 1.**
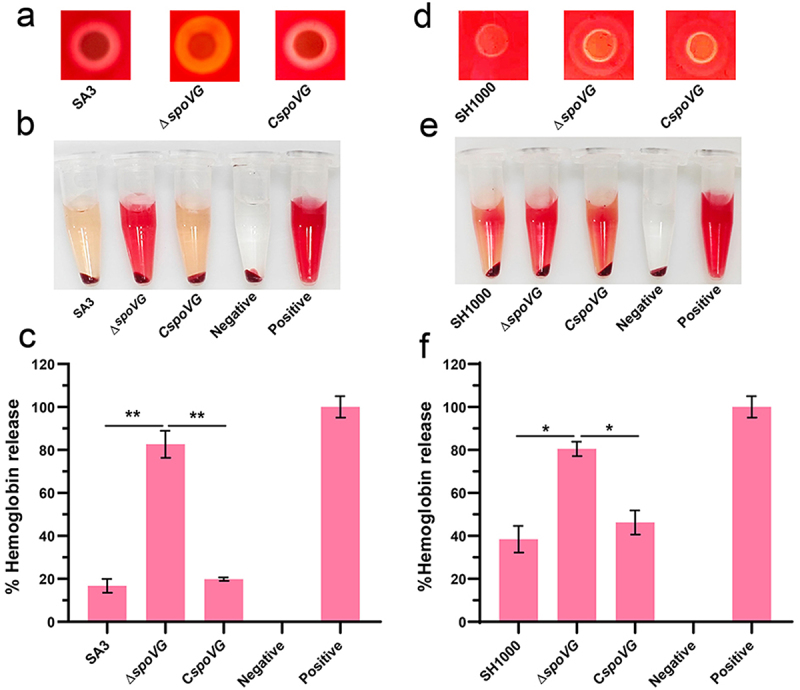
SpoVG negatively regulates the hemolytic activity of *S. aureus* SA3. (a) Hemolytic activities of the WT, *spoVG* mutant, and complemented strains were evaluated on SBA plates. (b) Haemolytic activities of the WT, *spoVG* mutant and complemented strains were determined by incubating samples with 5% sheep red blood cells. PBS and ddH_2_O were used as negative and positive controls, respectively. (c) The hemolytic activity of WT, *spoVG* mutant, and complemented strains was detected by measuring the absorbance value of the supernatant at OD_543_. (d) Hemolytic activities of the SH1000 and *spoVG* mutant and complemented strains were determined on SBA plates. (e) Hemolytic activities of the SH1000, *spoVG* mutant, and complemented strains were determined by incubating samples with 5% sheep red blood cells. (f) Hemolytic activities of the SH1000 and *spoVG* mutant and complemented strains were determined by measuring the absorption of supernatants at 543 nm. *,*p* < 0.05, **,*p* < 0.01, ***,*p* < 0.001.

Hemolytic activity is a key virulence trait of *S. aureus* that contributes to its pathogenicity [2]. To investigate whether SpoVG regulates the hemolytic activity of *S. aureus*, we assessed hemolysis in WT, *spoVG* mutant, and complemented strains using both qualitative and quantitative approaches. Sheep blood agar assays revealed that the *spoVG* mutant produced a significantly larger hemolytic ring than the WT strain, a restored phenotype that was observed in the complemented strain (Figure 1(a)). Quantitative hemolysis assays, performed by measuring OD_543_ in fresh sheep blood suspensions, further confirmed that *spoVG* deletion significantly increased hemolysis activity compared to the WT and C*spoVG* strains (Figure 1(b,c)).

To further substantiate the role of SpoVG in hemolysis regulation, we investigated its effect on hemolysis in SH1000 strains (a widely used laboratory model strain of *S. aureus)*. Consistent with our findings in SA3, deletion of *spoVG* in SH1000 resulted in significantly larger hemolytic zones on blood agar plates compared to the WT strain, whereas *spoVG* genetic complementation effectively restored hemolysis to WT levels (Figure 1(d)).

Quantitative hemolysis assays further confirmed this trend, as the *spoVG* mutant exhibited a higher OD_543_ value, indicative of increased erythrocyte lysis, while the complemented strain displayed hemolytic activity comparable to WT (Figure 1(e,f)). In this study, we demonstrate that SpoVG acts as a negative regulator of hemolysis in two *S. aureus* strains with distinct genetic backgrounds, highlighting its conserved function and crucial role in virulence regulation.

### SpoVG negatively regulates the expression of virulence genes

**Figure 2.**
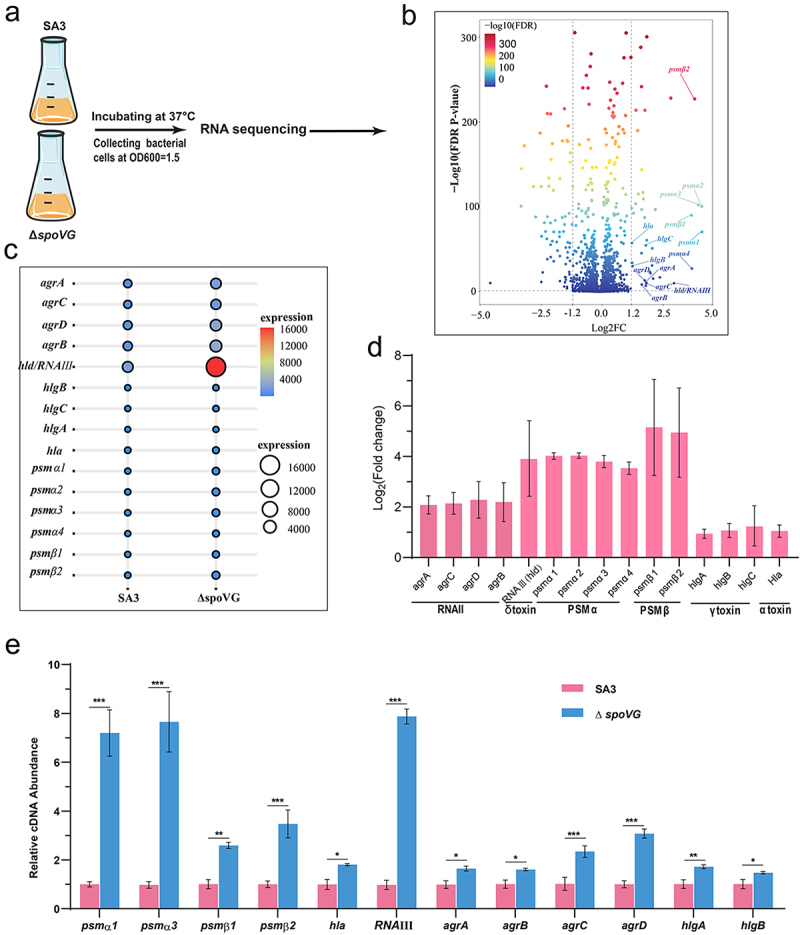
Transcriptional profiling of virulence-related genes of SA3 WT and *spoVG* mutant strain. (a) RNA sequencing conditions. (b) DEGs between SA3 and *spoVG* mutant strains, and virulence-related genes were identified. (c) This bubble plot shows the RPKM abundance of genes related to virulence between the SA3 and the *spoVG* mutant strain. (d) Fold changes in expression of virulence-associated genes. (e) RT-qPCR was used to verify the expression differences of virulence-related genes in the transcriptome. *,*p* < 0.05, **,*p* < 0.01, ***,*p* < 0.001.

To investigate how SpoVG modulates hemolytic activity in the SA3 strain, we conducted RNA sequencing (RNA-seq) to compare transcriptomes of the WT and *spoVG* deletion strains during exponential growth (OD₆₀₀ = 1.5) (Figure 2(a)). Transcript abundances were quantified for all 2,404 genes, and differentially expressed genes (DEGs) were identified to reveal functional distinctions between SA3 and Δ*spoVG*. In total, 218 DEGs were detected, including 85 upregulated and 133 downregulated genes in the Δ*spoVG* strain relative to the WT (log₂ fold change > 1; *p* < 0.05) (Figure 2(b)).

Notably, the deletion of *spoVG* resulted in a significant upregulation of all four core genes (*agrA*, *agrC*, *agrD*, *agrB*) and its effector regulator RNAIII. Additionally, the transcription levels of key with hemolysis- and virulence-associated genes, including *psmα* (encoding phenol-soluble modulins α), *psmβ* (encoding phenol-soluble modulins β), *hla* (encoding α hemolysin), and *hlgABC* (encoding γ hemolysin), were also significantly increased (Figure 2(c,d)). RT-qPCR analysis showed that the expression of *agrA*, *agrC*, *agrD*, *agrB*, *psmα*, *psmβ*, *hla*, and *hlgABC* was significantly increased in the *spoVG* mutant compared to the WT strain (Figure 2(e)), which is consistent with the RNA-seq results. Previous studies have demonstrated that AgrA directly interacts with the promoter regions of *psmα* and *psmβ*, thereby activating their transcription. Moreover, RNAIII enhances the expression of *hla* and *hlgABC* by binding to *hla* mRNA and the *hlgABC* promoter region, respectively. Therefore, we speculate that *spoVG* may negatively regulate the *agr* system, thereby inhibiting the virulence of the SA3 strain.

### Overexpression of spoVG decreased the hemolytic activity of *S. aureus*

**Figure 3.**
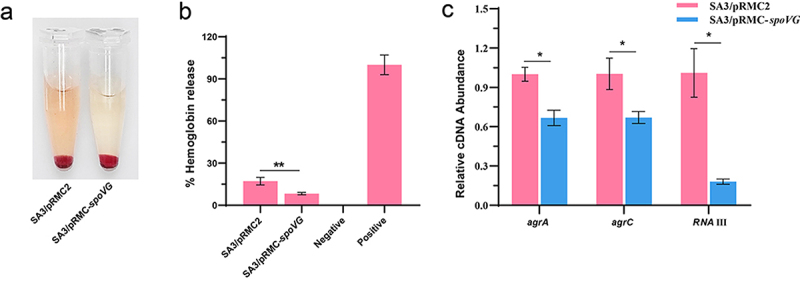
Overexpression of *spoVG* decreased hemolytic activity of *S. aureus*. (a) Hemolytic activities of the WT strains containing plasmids pRMC2 and pRMC-spoVG were determined by incubating samples with 5% sheep red blood cells. (b) The hemolytic activity of the WT strains containing plasmids pRMC2 and pRMC-*spoVG* was detected by measuring the absorbance value of the supernatant at OD_543_. (c) Overexpression of *spoVG* decreases transcription levels in the agr system. *, *p* < 0.05, **, *p* < 0.01, ***, *p* < 0.001.

To further evaluate the effect of *spoVG* on the hemolytic activity of the SA3 strain, we constructed a *spoVG*-overexpressing strain (SA3/pRMC-*spoVG*) and compared its hemolytic activity with that of the parental strain (SA3/pRMC2). Overexpression of *spoVG* significantly reduced hemolytic activity compared to the WT strain (Figure 3(a,b)). Furthermore, we examined the expression levels of genes involved in the agr system using RT-qPCR assays. Given that *agrDBAC* are co-transcribed, we selected *agrA*, *agrC*, and RNAIII as target genes for analysis. As shown in Figure 3(c), overexpression of *spoVG* significantly decreased the expression levels of the agr system compared to the WT strain. Meanwhile, we profiled *spoVG* expression across the full growth cycle and compared *agr* system expression between the *spoVG* mutant and the WT strain. *spoVG* expression progressively increased during growth, followed by a slight decline in the late logarithmic phase (Supplementary Fig. S1). For the expression levels of the genes encoding the *agr* system, *agr* expression in the *spoVG* mutant was comparable to the WT in the early logarithmic phase but was significantly upregulated as growth progressed (Supplementary Fig. S2). These data further support the notion that SpoVG negatively regulates the *agr* system, thereby affecting the hemolysis of the SA3 strain.

### SpoVG regulates the virulence of *S. aureus* through an agr-dependent pathway

**Figure 4.**
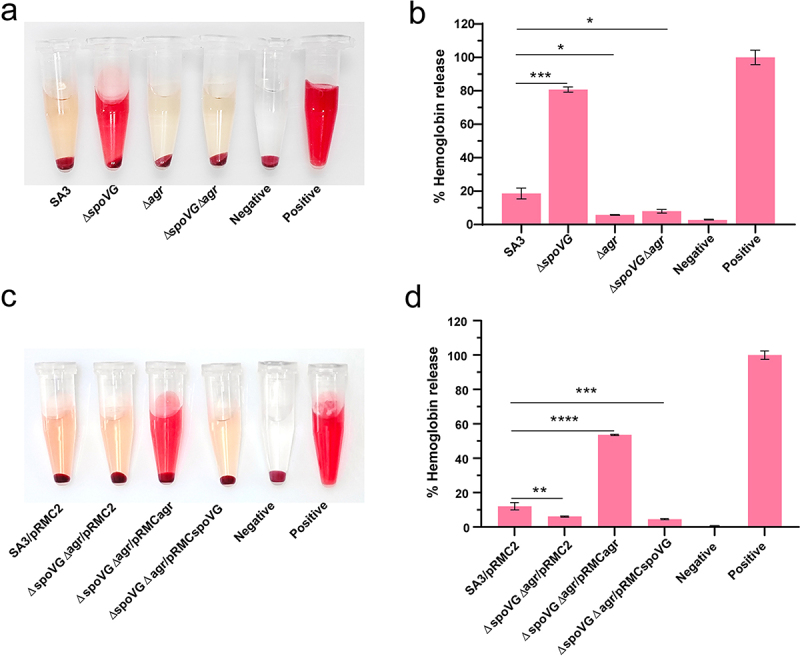
The effect of *spoVG* on the hemolytic activity of *S. aureus* is *agr*-dependent. Hemolytic activities of the strains SA3, Δ*spoVG*, Δ*agr*, Δ*spoVG*Δ*agr* (a) and SA3/pRMC2, Δ*agr*Δ*spoVG*/pRMC2, Δ*agr*Δ*spoVG*/pRMCagr, Δ*agr*Δ*spoVG*/pRMC*spoVG* (c) strains, the hemolytic activities were determined by incubating the target samples with 5% sheep erythrocytes, PBS (negative control), or ddH_2_O (positive control, 100% hemolytic activity). (b, d) the percentage of released hemoglobin (relative to the positive control) was determined by measuring the absorbance of supernatants at 543 nm. *, *p* < 0.05, **,*p* < 0.01, ***,*p* < 0.001, ****,*p* < 0.0001.

To further determine whether SpoVG regulates the virulence of *S. aureus* through an *agr*-dependent pathway, we constructed Δ*agr* mutant, Δ*agr*Δ*spoVG* double mutant, and complemented the double mutant with either *agr* or *spoVG* individually. Hemolytic activity was then assessed in these strains. No significant difference in the hemolytic activity was observed between the Δ*agr* mutant and the Δ*agr*Δ*spoVG* double mutant, both of which exhibited significantly lower hemolytic activity compared to the WT strain. In contrast, the Δ*spoVG* mutant displayed significantly higher hemolytic activity than both the Δ*agr* mutant and the Δ*agr*Δ*spoVG* double mutant (Figure 4(a,b)). Meanwhile, the Δ*agr*Δ*spoVG*/pRMC2 and Δ*agr*Δ*spoVG*/pRMCspoVG strains exhibited similar hemolytic activity, while the hemolysis in the Δ*agr*Δ*spoVG*/pRMC*agr* strain was significantly higher than in the Δ*agr*Δ*spoVG*/pRMC2 control (Figure 4(c,d)). These results suggest that the effect of SpoVG may play an important role on the virulence of *S. aureus*, which is an *agr*-dependent pathway.

### SpoVG directly binds to the promoter of *agr, psmα*, and *psmβ*

**Figure 5.**
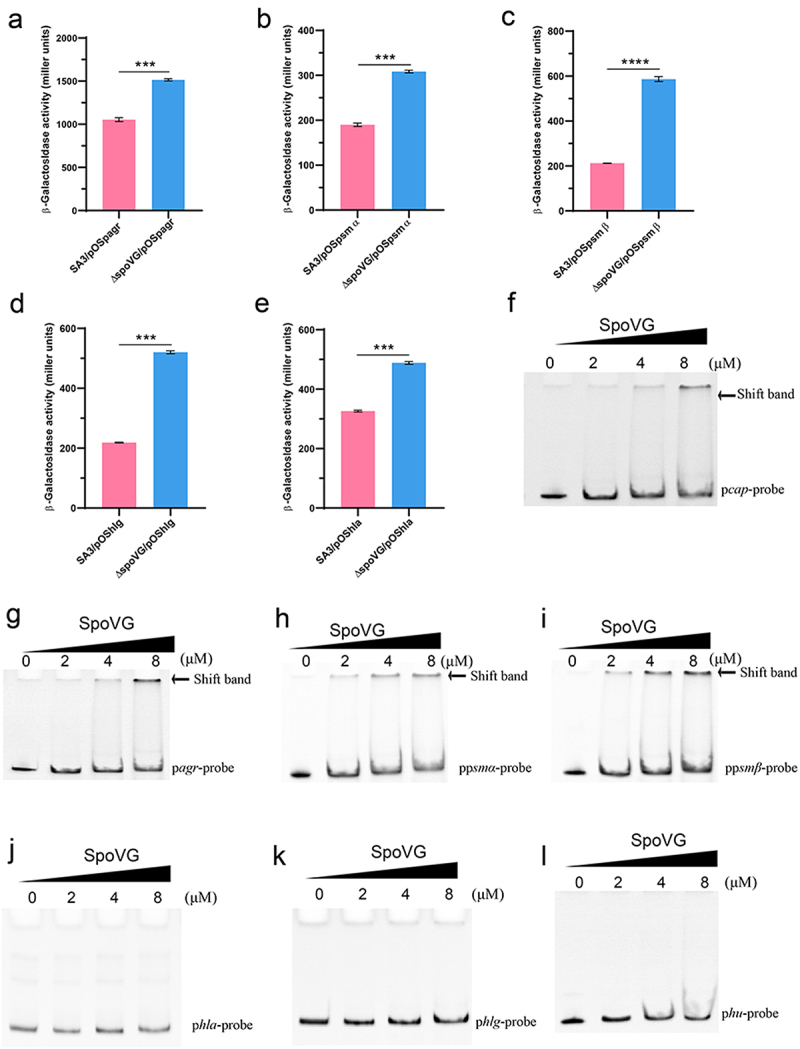
SpoVG specifically binds to the agr, p*smα*, and p*smβ* promoter regions. Promoter activity of *agr* (a), *psmα* (b), *psmβ* (c), *hlg* (d), *hla* (e) virulence gene. The p*cap* (f), p*agr* (g), p*psmβ* (h), p*psmα* (i), p*hla* (j), p*hlg* (k) and *hu* (l) DNA fragments were detected by EMSA with purified SpoVG protein, and then the concentrations of purified SpoVG protein were gradually increased, respectively, 0, 2, 4, and 8 μM, respectively. Where p*cap* is the positive control and *hu* is the negative control. *,*p* < 0.05, **,*p* < 0.01, ***,*p* < 0.001.

RT-qPCR and transcriptomic data confirmed that SpoVG negatively regulates the *agr* system and its downstream target genes. To further assess whether the regulatory effects of SpoVG on the *agr* operon, *psmα*, *psmβ*, *hla*, and *hlg* are mediated at the transcriptional level, we constructed a series of β-galactosidase reporter plasmids to quantify the promoter activities of these target genes. LacZ reporter assays revealed that the promoter activities of *agr*, *psmα*, *psmβ*, *hla*, and *hlg* were significantly increased in the *spoVG* mutant compared to the WT SA3, consistent with the transcriptomic and RT-qPCR results (Figure 5(a–e)).

To determine whether the altered transcription of *agr*, *psmα*, *psmβ*, *hla*, and *hlg* is directly regulated by SpoVG, we purified recombinant SpoVG-His6 and performed electrophoretic mobility shift assays (EMSA) using the corresponding promoter fragments. The results demonstrated that SpoVG retarded the mobility of the *agr*, *psmα*, and *psmβ* promoter fragments (Figure 5(g–i)). However, the *phla*, *phlg* promoter fragment was not retarded the mobility by the SpoVG protein (Figure 5(j,k)). These data suggest that SpoVG directly regulates the transcription of *agr*, *psmα*, and *psmβ* by binding their promoter regions, whereas *hla* and *hlg* are likely regulated through an indirect mechanism. The *cap* promoter served as a positive control (Figure 5(f)), and the *hu* promoter as a negative control (Figure 5(l)).

### SpoVG inhibited subcutaneous abscess model of mice of *S. aureus* virulence

**Figure 6.**
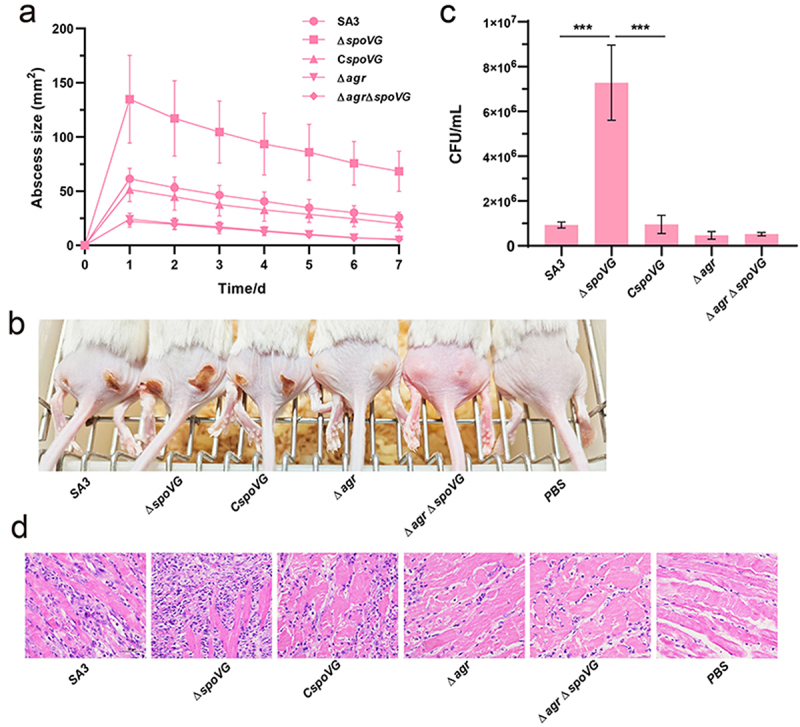
Deletion of *spoVG* enhances the virulence of *S. aureus* in a mouse subcutaneous abscess model. The mice were inoculated with 50 μl PBS containing 2.5 × 10^8^ CFU of the WT, *spoVG* mutant, *spoVG* complemented, *agr* mutant, and *agr spoVG* double mutant strains, or PBS alone as a control, in both flanks of the back by subcutaneous injection. (a) The abscess area was measured daily. (b) The photographic images of representative abscesses in mice 7 d after infection. (c) Determination of CFU in each abscess. (d) H&E staining of representative mouse abscesses. *,*p* < 0.05, **,*p* < 0.01, ***,*p* < 0.001.

The previous studies have demonstrated that Hla, Hlg, and PSMs play crucial roles in *S. aureus* skin and soft tissue infections. Given that our study demonstrated that SpoVG directly regulates the virulence of *S. aureus*, we employed a mouse subcutaneous abscess model to further investigate the role of SpoVG to the virulence of *S. aureus*. As shown in (Figure 6(a,b)), the abscess area induced by the Δ*spoVG* strain was significantly larger than that caused by the WT strain and the complemented strain, while the abscess areas of the Δ*agr* and Δ*agr*Δ*spoVG* strains were smaller. Furthermore, the bacterial load in the skin abscesses of mice infected with the Δ*spoVG* strain was significantly higher than that of the WT strain (Figure 6(c)). Tissue section analysis revealed that skin abscesses in mice caused by *spoVG* mutants exhibited more severe inflammation and leukocyte infiltration, as observed through hematoxylin and eosin (H&E) staining (Figure 6(d)). In summary, SpoVG negatively regulates the expression of virulence genes, thereby controlling the infection capacity of *S. aureus*.

## Discussion

As an opportunistic human pathogen, *S. aureus* can cause a variety of syndromes, including bacteremia, skin infections, and endocarditis [30,31]. These infections typically require the synergistic action of multiple virulence factors, for instance, the formation of skin infections necessitates the co-promotion of Hla and PSMs [32]. The treatment of *S. aureus* infections has become increasingly challenging due to the emergence of multidrug-resistant strains, and studies have demonstrated that *S. aureus* has a complex network of virulence regulations involving the Agr system, the Sar family, and several TCS [33,34]. Therefore, a clear understanding of the molecular mechanisms underlying bacterial virulence regulation is crucial for the prevention and treatment of *S. aureus* infections. However, our understanding of *S. aureus* remains incomplete, with many molecular mechanisms in virulence regulation still to be elucidated. Previous research has indicated that SpoVG plays a key role in arginine synthesis, antibiotic resistance, and metal ion tolerance in *S. aureus* [18,19,35], yet little is known about its involvement in the regulation of virulence. In this study, we found that SpoVG is involved in the virulence regulation of *S. aureus* through an Agr-dependent pathway and exerts an inhibitory effect on *S. aureus* virulence.

**Figure 7.**
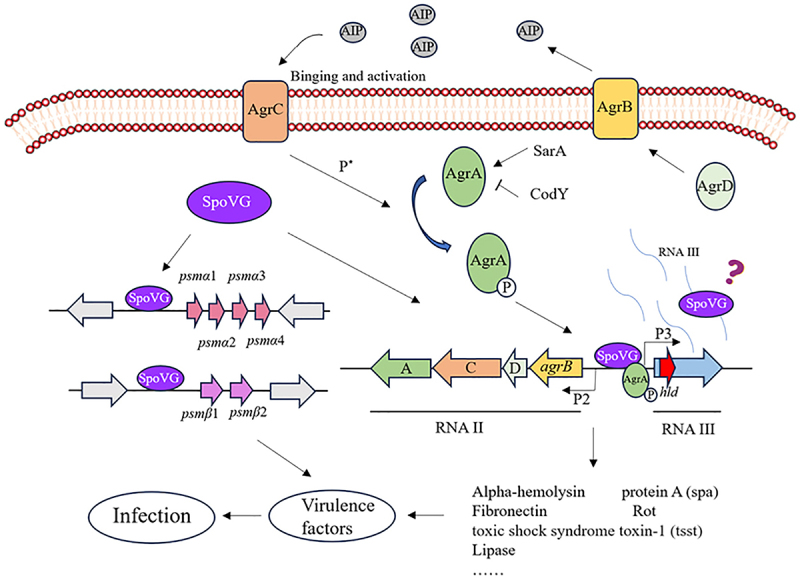
Schematic diagram of SpoVG-mediated regulation of *S. aureus* virulence.

Previous research has demonstrated that the Agr quorum-sensing system modulates target gene expression in a cell-density-dependent pathway and is additionally regulated by other transcriptional factors, including SarA, CodY, and MgrA [36,37]. Our findings indicate that SpoVG influences the virulence of *S. aureus* by regulating the expression level of Agr system, which contributes novel insights into the virulence regulatory network in *S. aureus*. Previous studies in certain Gram-positive bacteria have reported that SpoVG not only directly binds to DNA but also regulates target gene expression through direct interaction with RNA [38,39]. However, to date, no evidence has been found supporting the notion that SpoVG directly interacts with RNA to regulate target genes in *S. aureus*. RNAIII serves as a pivotal effector in the Agr system, playing a critical role in the expression and regulation of several virulence factors. Moreover, previous studies in *S. aureus* have shown that only AgrA directly binds the P3 promoter to regulate RNAIII transcription. We therefore propose three potential mechanisms by which *spoVG* may regulate RNAIII expression: (i) indirectly, via modulation of *agr* transcription; (ii) directly, by binding the P3 promoter to control RNAIII transcription; and (iii) post-transcriptionally, by binding RNAIII itself to modulate virulence gene expression. These hypotheses remain to be tested and will be the focus of future studies aimed at elucidating the SpoVG regulatory network in virulence control (Figure 7).

In *S. aureus*, certain regulatory mechanisms and functions exhibit strain specificity. For example, the Rsp transcriptional regulators of AraC/XylS family exert distinct virulence regulatory effects in the N315 and Newman strains [23,40]. To investigate whether the regulatory manner of *spoVG* on virulence is conserved in strains with different genetic backgrounds, we evaluated the hemolytic phenotype of *spoVG* mutants in SH1000 strains. The results revealed that the *spoVG* mutant exhibited significantly enhanced hemolytic activity compared to the SH1000 strain, and this phenotype was restored by genetic complementation. This finding is consistent with our observations in the SA3 strain, suggesting that *spoVG* may be conserved in regulating *S. aureus* virulence in different genetic backgrounds. Previous studies have demonstrated that SpoVG regulates the virulence of *S. aureus* N315 by directly modulating Rot, a transcriptional regulator of the Sar family [20]. However, Rot is negatively regulated by RNAIII of the Agr system, suggesting that SpoVG may bypass the Agr system to directly regulate Rot in the N315 strain. This alternative regulatory mechanism may be linked to the natural inactivation of the Agr system in N315. These findings underscore the complexity of *S. aureus* virulence regulation and emphasize the importance of strain-specific genetic factors in determining virulence control.

## Supplementary Material

Author Checklist.pdf

figure S2.jpg

figure S1.jpg

## Data Availability

The raw RNA-seq data have been deposited in the National Center for Biotechnology Information (NCBI) Gene Expression Omnibus database under the SRA accession number **PRJNA1255731** (https://www.ncbi.nlm.nih.gov/sra/PRJNA1255731). All other data supporting the findings of this study are available within the Science Data Bank: https://doi.org/10.57760/sciencedb.24885, reference [41].
