# Supplementary figures and images for: The global regulator SpoVG modulates Staphylococcus aureus virulence through Agr-dependent pathways
Source: Virulence. 2025 Sep 15;16(1):2561827. doi: 10.1080/21505594.2025.2561827 (PMC12445516; doi:10.1080/21505594.2025.2561827)

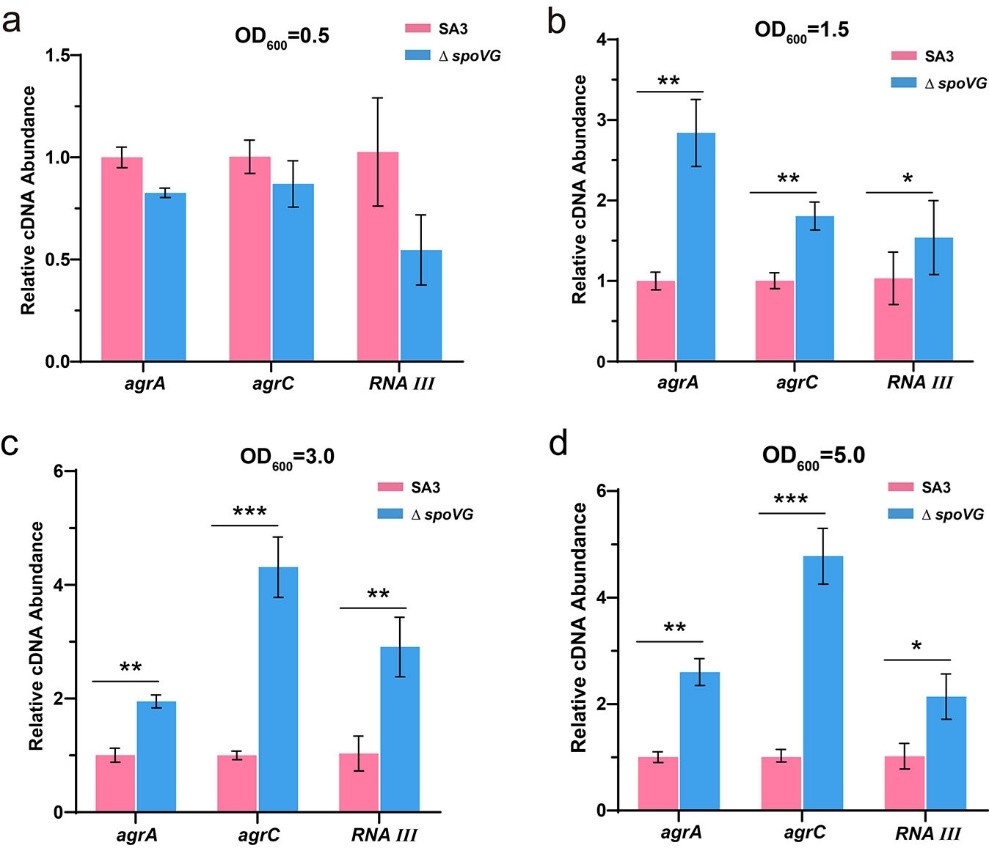

Supplement: figure S2.jpg [file KVIR_A_2561827_SM3139.jpg]

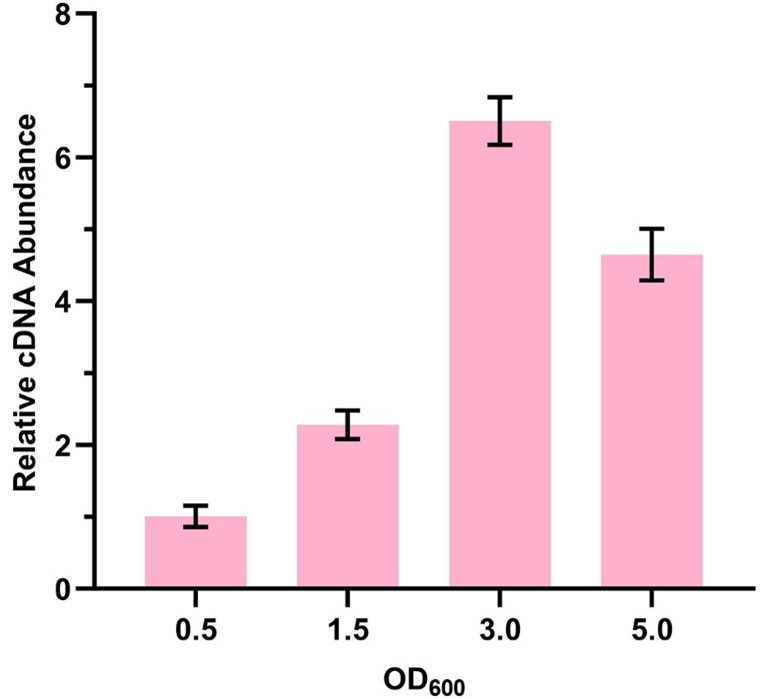

Supplement: figure S1.jpg [file KVIR_A_2561827_SM3138.jpg]
